# Analysis of Global Sumoylation Changes Occurring during Keratinocyte Differentiation

**DOI:** 10.1371/journal.pone.0030165

**Published:** 2012-01-23

**Authors:** Phillip R. Heaton, Andres Santos, Germán Rosas-Acosta, Van G. Wilson

**Affiliations:** Department of Microbial and Molecular Pathogenesis, College of Medicine, Texas A&M Health Science Center, College Station, Texas, United States of America; University of South Florida College of Medicine, United States of America

## Abstract

Sumoylation is a highly dynamic process that plays a role in a multitude of processes ranging from cell cycle progression to mRNA processing and cancer. A previous study from our lab demonstrated that SUMO plays an important role in keratinocyte differentiation. Here we present a new method of tracking the sumoylation state of proteins by creating a stably transfected HaCaT keratinocyte cell line expressing an inducible SNAP-SUMO3 protein. The SNAP-tag allows covalent fluorescent labeling that is denaturation resistant. When combined with two-dimensional gel electrophoresis, the SNAP-tag technology provides direct visualization of sumoylated targets and can be used to follow temporal changes in the global cohort of sumoylated proteins during dynamic processes such as differentiation. HaCaT keratinocyte cells expressing SNAP-SUMO3 displayed normal morphological and biochemical features that are consistent with typical keratinocyte differentiation. SNAP-SUMO3 also localized normally in these cells with a predominantly nuclear signal and some minor cytoplasmic staining, consistent with previous reports for untagged SUMO2/3. During keratinocyte differentiation the total number of proteins modified by SNAP-SUMO3 was highest in basal cells, decreased abruptly after induction of differentiation, and slowly rebounded beginning between 48 and 72 hours as differentiation progressed. However, within this overall trend the pattern of change for individual sumoylated proteins was highly variable with both increases and decreases in amount over time. From these results we conclude that sumoylation of proteins during keratinocyte differentiation is a complex process which likely reflects and contributes to the biochemical changes that drive differentiation.

## Introduction

Cell survival, growth, and differentiation depend in large part on the cell's ability to respond to a wide variety of stimuli. Often times these responses are needed rapidly, and after the response is properly carried out the cell must be able to return to its pre-altered state. Rapid regulation of such response is often achieved through the reversible post translational modification of proteins. Numerous means of post-translationally modifying a protein exist and some of the best characterized modifications are those that add a small chemical moiety to a protein such as phosphorylation, acetylation, and glycosylation. Post translational modifications can also include the addition of a small protein or peptide sequence to a protein as is the case with ubiquitin and other ubiquitin-like modifiers (UBLs). UBLs are a family of proteins that include SUMO, Nedd8, ISG15, URM1, ATG8, ATG12, FAT10, FUB1, UFM1, and UBL5 [1]. Ubiquitin is the best characterized of these protein modifications while SUMO (Small Ubiquitin-like Modifier) is the most studied of the remaining UBLs.

The Small Ubiquitin-like MOdifier (SUMO) was co-discovered by four different groups in 1996, and understanding of the protein's role in the cell has grown tremendously since its identification. SUMO, an 11 kD protein, is added to specific lysine residues in its target proteins, usually within the consensus sequence of ΨKXD/E (where Ψ is a hydrophobic residue, K is the target lysine residue, X is any amino acid, and D/E is aspartic or glutamic acid), although recent studies have also elucidated roles for modification at noncanonical lysine residues [2], [3], [4]. SUMO exhibits about 18% amino acid sequence homology to ubiquitin though their overall three dimensional structures are almost identical [5]. Currently there are four SUMO isoforms with SUMO1, 2, and 3 being the most prominent as SUMO4 is restricted to certain cell types. SUMO1 shares about 48% amino acid homology to SUMOs 2 and 3 while SUMOs 2 and 3 have roughly 92% amino acid similarity to one another [6]. SUMO conjugation involves a series of enzymatic reactions that eventually lead to the modification of target proteins and closely resembles the mechanism by which ubiquitin is attached to its targets. SUMO is first translated into a precursor protein that is inactive until it is cleaved by SUMO proteases known as SENPs. The SENPs are cysteine proteases and act to expose a C-terminal diglycine motif on SUMO that is needed for the remainder of the enzymatic steps; SENPS also remove SUMO from modified proteins thereby desumoylating modified proteins [7]. Once matured, SUMO is activated by the heterodimeric E1 enzyme in an ATP dependent manner. This enzyme is comprised of two subunits, SAE1 and SAE2, with SAE2 forming a thioester bond with SUMO and therefore providing the active site. SUMO is subsequently transferred to the SUMO conjugating enzyme, Ubc9, through the formation of another thioester bond. In the final step of the enzymatic process, SUMO is transferred to the target protein where it forms an isopeptide bond with the ε-amino group of the target lysine [8]. This final step is a departure from what is seen in ubiquitination as an E3 ligase is not absolutely required for sumoylation of proteins [9]. However, the presence of a SUMO E3 ligase can make the addition of the SUMO moiety to a target protein more efficient and/or affect substrate specificity.

Since the discovery of SUMO many proteins have been shown to be SUMO modified, and the functional significance of SUMO modifications has been increasingly broad; SUMO is now known to play roles in cell cycle regulation [10], transcriptional repression [11], and differentiation [12]. For keratinocytes, differentiation is linked to a calcium gradient, and our lab recently showed that SUMO plays a role in calcium induced keratinocyte differentiation [13]. Over the course of keratinocyte differentiation there was increased expression of the sumoylation enzymes and of SUMO2/3, leading to increased sumoylation of some host proteins over time. These results have been independently validated in both HaCaTs and primary cells [14]. Inhibition of the sumoylation system using Adenovirus Gam-1 protein prevents proper differentiation of keratinocytes, indicating that the changes in levels of the sumoylation components and the modification of substrates is functionally important. However, our prior study only looked at total sumoylated proteins on a 1D gel and did not attempt to explore the dynamics of incorporation of the individual SUMO isoforms into cellular substrates. The current study focuses on SUMO3 modification of proteins during keratinocyte differentiation by using 2D gel electrophoresis of whole cell lysates and detection of SUMO3 modified proteins using a novel SNAP-tag SUMO3 fusion protein. The SNAP moiety is a protein tag derived from the DNA repair enzyme O^6^-alkylguanine-DNA alkyltransferase which reacts with benzyl purines and benzyl pyrimidines [15]. Fluorophores based on these substrates form covalent adducts with the SNAP active site resulting in a strong fluorescent signal [16], [17]. Unlike GFP-based tags, the signal from the fluorophore bound to the SNAP moiety is stable to denaturation, and this allows protein isolation under stringent denaturing conditions which inactivates SUMO proteases and maintains substrates in their sumoylated form. We recently utilized the SNAP-SUMO3 approach to demonstrate the papillomavirus E6 oncoprotein causes alterations in the sumoylation pattern of HaCaT keratinocyte cells [18]. In this report we now show that the SNAP-SUMO technology can be used to follow and catalog dynamic changes in the SUMOeome temporally as keratinocyte differentiation proceeds. This is a novel technique that can be applied to any dynamic process and will greatly facilitate visualization of pertinent sumoylation targets for subsequent identification and characterization.

## Results

### Induction and optimization of SNAP-SUMO3

Previous studies in our lab showed a correlation between keratinocyte differentiation and sumoylation [13]. During calcium-induced HaCaT keratinocyte differentiation, there is an increase in the expression levels of the sumoylation enzymes SAE2/1 and Ubc9, of the modifiers SUMO2/3, as well as changes in the levels of at least some sumoylated substrates. The increased expression of SUMO2 during keratinocyte differentiation has been also reported by other groups in both HaCaTs and primary keratinocytes [14]. We also demonstrated the functional importance of sumoylation for keratinocyte differentiation by using the adenoviral Gam1 protein to abrogate the sumoylation machinery which in turn prevented keratinocytes from differentiating properly. The goal of our current study was to determine whether these changes in the expression of the sumoylation components during keratinocyte differentiation resulted in specific changes in the pool of sumoylated proteins at different stages of the differentiation process. Such a global analysis of sumoylation dynamics over the course of a multi-day cellular process has not previously been performed.

**Figure 1:**
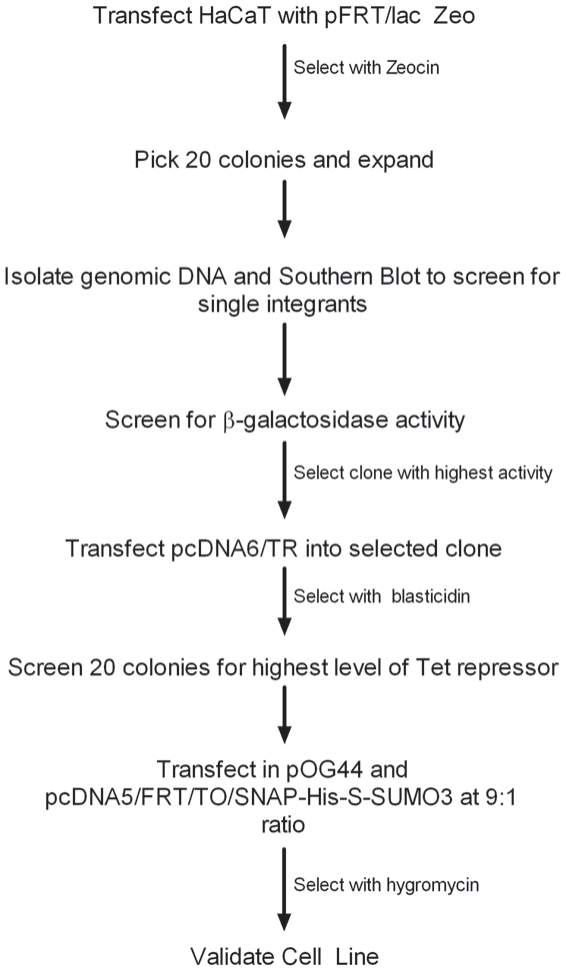
Work flow for creating cell lines containing SNAP-tagged SUMO3. The HaCaT SNAP-SUMO3 cell line was generated from HaCaT cells and was validated after selection with hygromycin and blasticidin.

**Figure 2:**
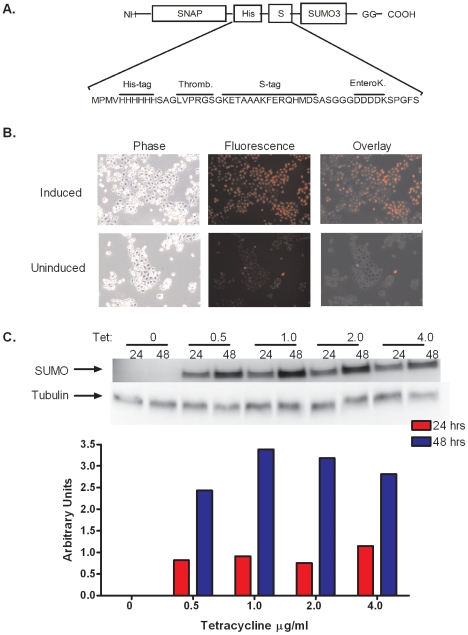
HaCaT-SNAP SUMO3 cells express SNAP-tag and are capable of both induction and labeling. (**A**) Schematic of the SNAP-SUMO3 sequence showing the order of the SNAP, His, and S tags attached to the N terminal of SUMO3. (**B**) HaCaT SNAP-SUMO3 cells were plated into induced or uninduced groups and labeled with SNAP TMR-Star followed by visualization with phase contrast or fluorescent microscopy. (**C**) Induction optimization was done using 0, 0.5, 1, 2 and 4 µg/ml tetracycline, and samples were collected either 24 or 48 hours post induction. Visualization of SUMO3 was done by blotting and detection with S protein conjugated to HRP. Free SNAP-SUMO3 was normalized to tubulin to determine the optimal concentration of tetracycline, and the results of the representative experiment shown are quantitated in the graph.

To accurately detect the dynamic state of protein sumoylation in differentiating keratinocytes we developed a new methodology for directly visualizing sumoylated proteins. This new approach involved constructing a cell line expressing a SNAP-tagged SUMO3 using the Invitrogen Flp-n T-REx system via the strategy depicted in Fig. 1. The resulting cell line contained a single extra copy of the SUMO3 gene modified to include coding sequences for three epitope tags at its N terminus: the SNAP, His, and S tags (Fig. 2A). The 20 kDa SNAP tag allows the covalent in vivo labeling of the SNAP-His-S-SUMO3 protein (subsequently referred to as SNAP-SUMO3) and consequently the labeling of SUMO3 modified substrates. The addition of the His and S tags allow for affinity purification of the modified proteins if needed. After generating the cell line we tested to ensure that the SNAP-tagged SUMO was expressed and capable of being detected in cells. Fig. 2B shows that nearly 100% of the cells induced with tetracycline show labeling within the cells, which is in stark contrast to the uninduced cells which show very little labeling. These results demonstrate that expression of the SNAP-SUMO3 protein can be induced and that the SNAP moiety can be labeled in vivo. To determine optimal induction conditions for the SNAP-tag SUMO3, cells were exposed to varying levels of tetracycline for either 24 or 48 hours. Cells were then lysed and SNAP-tagged SUMO3 was detected using S protein conjugated to HRP. Free SUMO3 was evaluated and normalized to tubulin as the conjugated species of SUMOs produce a complex pattern that proved difficult to normalize. Fig. 2C presents a representative experiment and shows that the optimal concentration for induction is 1 µg/ml of tetracycline for 48 hours. Induction for 24 hours did not provide enough time for maximal expression and using higher tetracycline concentrations did not improve SUMO3 expression and reproducibly caused a slight decrease in the SUMO3 levels. In addition to demonstrating induction, this experiment showed that the molecular weight of the free SUMO3 detected with the S protein-HRP conjugate was 37 kDa as predicted for a SNAP-His-S-SUMO3 fusion protein. This same band could also be detected by anti-His (not shown). These combined results indicate that this cell line correctly expresses an inducible SNAP-His-S-SUMO fusion protein, subsequently referred to as SNAP-SUMO3.

### Expression of SNAP-SUMO 3 does not affect cell cycle distribution, but changes growth characteristics

**Figure 3:**
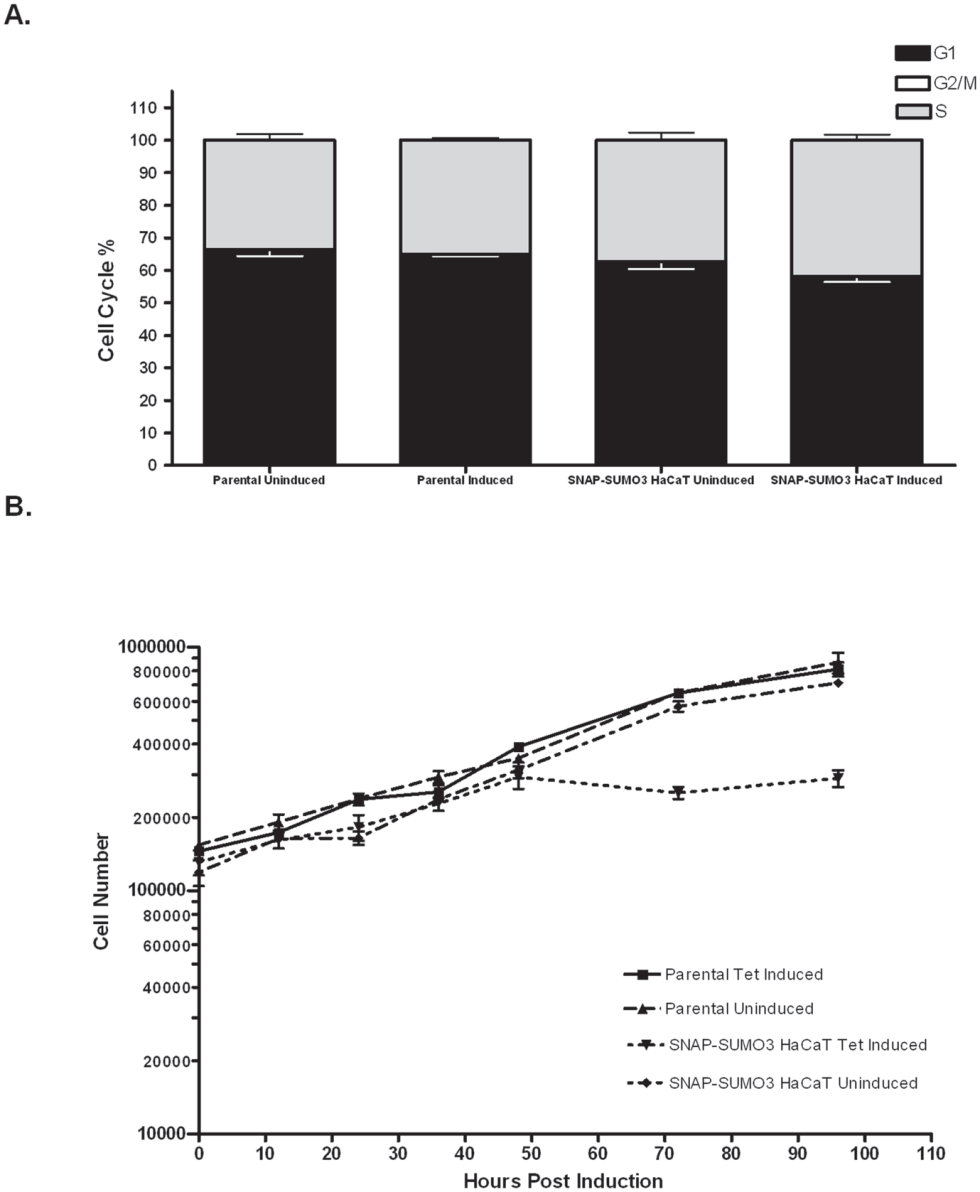
SNAP-SUMO3 expression does not affect cell cycle distribution, but does slow cell doubling. (**A**) HaCaT cells were plated and divided into uninduced and induced groups. Induction of SNAP-SUMO3 was for 48 hours with 1.0 ug/ml of tetracycline. After 48 hours the cells were analyzed by FACS to determine cell cycle distributions. The experiment was performed three times and the results shown are the mean and standard error of the mean. (**B**) SNAP-SUMO3 HaCaT cells and parental HaCaT FRT/TR#8 cells were grown and induced as in (**A**). Triplicate cultures were prepared and counted each day and averages were taken. Error bars represent mean ± standard deviation.

SUMO plays a role in a number of cellular processes and its effects on cell cycle and growth are well documented [19], [20], [21]. As our cell line efficiently expresses SUMO3 after induction with tetracycline, we wanted to ensure that the cell cycle and growth characteristics were not perturbed. We employed FACS analysis (Fig. 3A.) to examine the cell cycle distribution after expression of SNAP-SUMO3. The parental cell line that does not have the SNAP-SUMO3 was used as a control to gauge the effects of tetracycline. Neither tetracycline exposure nor expression of SNAP-SUMO3 affected the cell cycle distribution (Fig. 3A and Fig. S1). It is interesting to note that very few of the cells were found to be in G2/M phase of the cell cycle most likely due to the possible shorter duration of this phase of the cell cycle in the HaCaT line. The next aspect of our evaluation of SUMO expression was to determine the effects of SNAP-SUMO3 expression on cell doubling (Fig. 3B). Cells were plated and counted every 24 hours and an average was taken for each time point. Interestingly, while induction of SNAP-SUMO3 did not affect cell cycle distribution in the short term, it appeared to halt cell doubling between 48 and 72 hours post induction. It is known that over expression of SUMO induces senescence in some cells and this might explain the observed lack of cell doubling after 48 hours of SUMO3 induction. To determine whether expression of SNAP-SUMO3 caused senescence we performed an assay for senescence associated β-galactosidase activity (Fig. S2 and Materials and Methods S1). Human foreskin fibroblasts (HFF) infected with the Towne laboratory strain of human cytomegalovirus (CMV) at an MOI of 5 served as the positive control while mock infected HFFs served as the negative control. HaCaT SNAP-SUMO3 cells were divided into tetracycline-induced and uninduced groups with three plates for every time point, and the assay was conducted every 24 hours for 72 hours. Figure S2 shows that after 72 hours in the presence of tetracycline the HaCaT SNAP-SUMO3 cells started to become positive for senescence associated β-galactosidase activity, suggesting that the lack of population doubling after 48 hours is associated with senescence. Since no senescence associated β-galactosidase activity was observed at 48 hours post Tet-induction, all subsequent experiments were done within this 48 hour time frame to minimize senescence effects while still providing sufficient SNAP-SUMO3 induction for effective detection of sumoylated substrates. Additionally, for the subsequent differentiation experiments all time points, including the time 0 sample, were induced for SNAP-SUMO3 expression for 48 hrs prior to harvest so that any influence of potential pre-senescent changes should be equivalent in all samples.

### HaCaT SNAP-SUMO3 cells differentiate like normal HaCaTs

**Figure 4:**
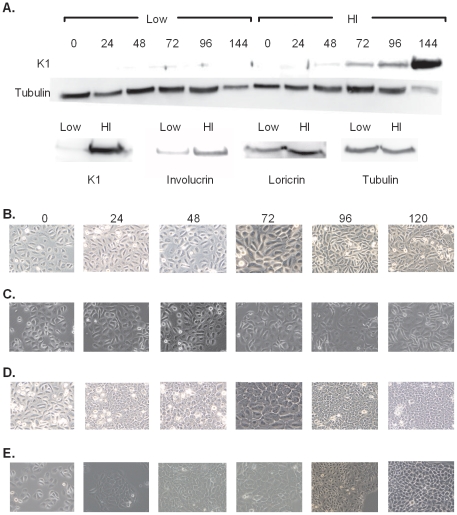
HaCaT SNAP-SUMO3 cells display normal biochemical and physical characteristics of keratinocyte differentiation. (**A**) SNAP-SUMO3 HaCaT cells were plated and induced to differentiate with 2.38 mM calcium. SNAP-SUMO3 induction was started 48 hours prior to harvest for each time point. Samples were collected at 24 hour intervals from 0–144 hours and were analyzed on 8% gels followed by Western blotting for K1, involucrin, loricrin, and tubulin. The upper figure shows a time based progression of K1 expression between differentiating (HI) and basal (Low) cultures. The lower panel shows the differences at 144 hours for K1, involucrin, and loricrin expression between high and low calcium SNAP-SUMO3 HaCaT cells. (**B through E**) Time course comparison of the physical morphology of basal (**B and C**) and differentiating (**D and E**) SNAP-SUMO3 HaCaT keratinocytes (B and D) versus parental HaCaT cells (C. and E) using phase microscopy. Magnification is 200× except for the 72 hour samples which were at 320×.

Prior to analysis of sumoylation during differentiation it was necessary to ensure that the HaCaT SNAP-SUMO3 cell line behaved like normal HaCaTs with regards to marker expression and morphology when induced to differentiate with calcium. HaCaT SNAP-SUMO3 cells were plated so that the cells would be between 80 and 85% confluent at the time of harvest or image capture. Cells were divided into basal and differentiating groups, and differentiation was initiated by addition of media containing 2.38 mM calcium. Keratin 1 was used as a marker of keratinocyte differentiation as it is expressed in the spinous and granular layers of the epidermis. Figure 4A shows that upon addition of high calcium medium K1 protein appeared at 72 hours and continuously increased to 144 hours post differentiation. Basal cells did not exhibit any expression of K1 throughout the time course of this study (Fig. 4A, Low). The kinetics of K1 expression in the SNAP-SUMO3 HaCaT line are equivalent to that observed for the parental HaCaT cells (Fig. S3; [18]). In addition to K1, two other markers of keratinocyte differentiation, involucrin and loricrin, were also induced by calcium, so the biochemical markers of differentiation appear normal in this cell line further validating the use of this SNAP-SUMO3 cell line as a keratinocyte model. Morphologically, SNAP-SUMO3 HaCaT (Fig. 4D) and HaCaT (Fig. 4E) cells treated with high calcium quickly became more cuboidal and more tightly packed with the presence of tight junctions, while untreated HaCaT SNAP-SUMO3 cells (4B) displayed a more loosely connected phenotype, with the cells having a more spindle-like appearance characteristic of basal, undifferentiated HaCaTs (Fig. 4C). We previously reported a slight delay in differentiation morphology with the SNAP-SUMO3 HaCaT line [18], but this effect is not consistent and there appears to be no significant different differentiation kinetics. Taken together the marker expression levels, the morphological features, and the kinetics of changes observed after calcium induction indicated that the SNAP-SUMO3 line was identical to the parental HaCaT cells, therefore demonstrating that SNAP-SUMO3 cell line is not appreciably altered in its keratinocyte differentiation properties.

Lastly, it was also important to ensure that the cellular localization of the SNAP-SUMO3 protein mirrored that of native SUMO3. Endogenous SUMO3 in HEK293 cells is found primarily in the nucleus with both diffuse distribution and accumulation in nuclear bodies; unlike SUMO1, SUMO3 is also present in the cytoplasm and exhibits diffuse distribution in the cytoplasm [22]. Confocal microscopy (Materials and Methods S1) demonstrated that endogenous SUMO3 in parental HaCaT cells and the uninduced SNAP-SUMO3 HaCaT cells displayed a distribution similar to that reported for 293 cells (Fig. S4, D–I). Importantly, SNAP-SUMO3 expressed in the stable SNAP-SUMO3 HaCaT cell line localized like endogenous SUMO3 and was preferentially located in the nucleus, with the punctate accumulation within nuclear bodies (Fig. S4, A–C); some of the nuclear deposit of SNAP-SUMO3 appeared larger than for endogenous SUMO3 which may reflect higher expression levels of the fusion protein. As reported for endogenous SUMO3, SNAP-SUMO3 also exhibited diffuse labeling in the cytoplasm. The ability of SNAP-SUMO3 to localize in a similar fashion to endogenous SUMO3 further supports the utility of this cell line as a model system for observing sumoylation in the keratinocytes.

### SNAP-SUMO3 is functional for conjugation to substrates

**Figure 5:**
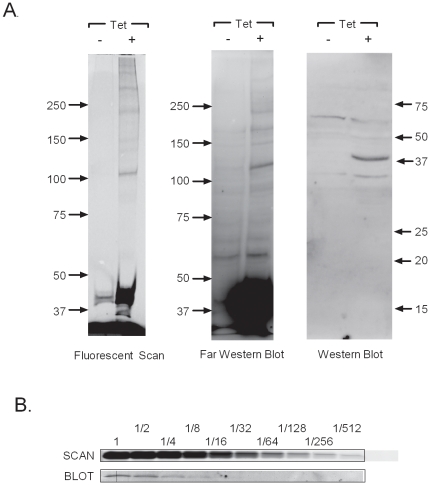
SNAP-SUMO3 is functional and capable of being detected in gel. (**A**) SNAP-SUMO3 HaCaT cells were divided into induced (+lanes) and uninduced (−lanes) groups, and the induced groups were treated for 48 hours with 1.0 ug/ml of tetracycline. At 48 hours post Tet addition, cells were labeled with SNAP-cell DAF, lysed with 4× sample buffer, and electrophoresed on 6% (left and middle panels) or 15% (right panel) SDS polyacrylamide gels. The gels were either imaged using a Fuji FLA-5100 (Fluorescent Scan) or were transferred to a PVDF membrane and detected using S-protein conjugated to HRP (Far Western Blot panel) or anti-SUMO2/3 (Western Blot panel). (**B**) Induced extracts prepared as in (**A**) were subjected to 2-fold serial dilutions, electrophoresed on an 8% SDS polyacrylamide gel, and then free SNAP-SUMO3 was detected either by fluorescent scan or by immunoblotting with anti-SUMO3.

Before conducting large scale experiments that examine sumoylation patterns in differentiating keratinocytes by 2D gel electrophoresis we wanted to make sure that SNAP-SUMO3 labeled in vivo is efficiently incorporated into cellular substrates. Uninduced and tetracycline induced cells were labeled with SNAP-Cell DAF and lysed with 4X sample buffer. Parallel samples were run on 6% gels and either visualized for fluorescence directly using the Fuji-FLA 5100 (Fig. 5A, left panel) or transferred to a PVDF membrane and detected using S-protein conjugated to HRP (Fig. 5A, middle panel) or anti-SUMO2/3 antibody (Fig. 5A, right panel). In the induced samples, both fluorescence and the S-protein blot revealed a major band at the predicted molecular weight of free SNAP-His-S-SUMO3 (37 kDa). In addition, the induced samples had an array of higher molecular weight species that is consistent with covalent attachment of SNAP-SUMO3 to multiple cellular substrates. As expected, in the uninduced samples the amount of the free SNAP-His-S-SUMO3 protein was greatly reduced consistent with the very limited background expression observed in Fig. 2B. Detection of SNAP-SUMO3 through fluorescent labeling showed less background (compare the Tet minus lanes in each panel) while proving to be more sensitive when detecting high molecular weight conjugates above 250 kDa. To visualize endogenous SUMO2/3, the Western blot (Fig. 5A, right panel) samples were run on a higher percentage gel. Under these cell growth and immunoblotting conditions the SNAP-SUMO3 protein was clearly visible while endogenous SUMO2/3 was usually not detectable. The low expression of endogenous SUMO3 precluded a quantitative comparison of SUMO3 versus SNAP-SUMO3 expression levels, but these results did indicate that even the single-copy SNAP-SUMO3 cassette produced significantly more protein than the endogenous SUMO3 gene in HaCaT cells. Fortunately, the results in Figures 2, 3, 4 suggest that transient expression (48 hrs or less) of this level of SNAP-SUMO3 has no deleterious effects on cell phenotype or growth properties. However, the possibility of artifactual sumoylation of some substrates due to elevated SNAP-SUMO3 levels cannot be excluded and would need to be addressed on a substrate by substrate basis.

To further evaluate the relative sensitivity of fluorescent detection, we directly compared 2-fold serial dilutions of SNAP-SUMO3 extracts by fluorescent scanning versus immunoblotting with an anti-SUMO3 antibody (Fig. 5B). The free SNAP-SUMO3 band was detectable down to a 1∶512 dilution in the fluorescent scan, while the immunoblot only detected SNAP-SUMO3 to a 1∶8 dilution. We conclude from the results in Fig. 5A and 5B that in vivo labeled SNAP-SUMO is functional for conjugation to substrates, that in gel detection of sumoylated proteins is possible, and that the sensitivity of detection is significantly better than immunoblotting with this particular SUMO3 antibody. In addition, detection of SUMO3 via SNAP labeling avoids the need for protein transfer to membranes that may result in loss or non-quantitative transfer of some proteins.

### SUMO3 substrates change dynamically during HaCaT differentiation

**Figure 6:**
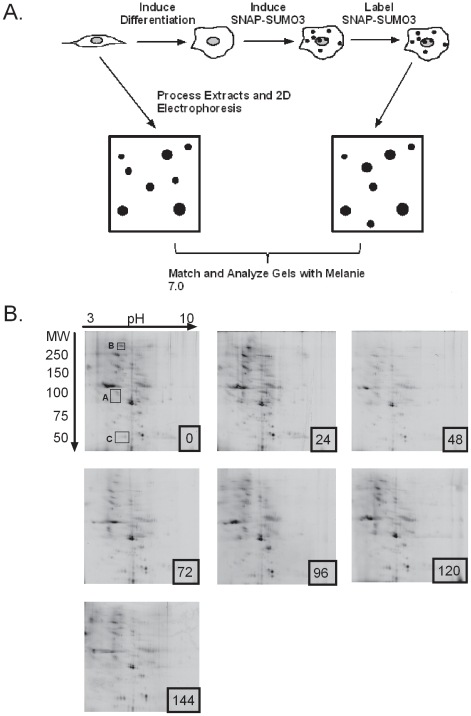
Kinetic analysis of sumoylation dynamics during keratinocyte differentiation. (**A**) Schematic representation of the procedure to evaluate sumoylation dynamics during the time course of keratinocyte differentiation. For each time point, cells are plated so that they are approximately 80% confluent at the time of harvest. Medium containing 2.38 mM calcium is used to induce differentiation at time 0 and cells are maintained for 144 hours post-induction of differentiation. At 48 hours prior to harvest for each time point, SNAP-SUMO3 is induced with tetracycline. At one hour prior to lysis cells are incubated with the fluorophore to label SNAP-SUMO3. Cells are lysed in 2D sample preparation buffer followed by 2D gel electrophoresis. Gels are then analyzed using the Melanie software to match and quantitate spots for the 0 time sample (Basal) versus each differentiated time point (Differentiated). (**B**) Representative set of gels from time zero (basal) to 144 hours post-induction of differentiation. Samples for each gel were processed and run as in Materials and Methods then the gels were scanned by a Typhoon Trio variable mode imager. For the quantitative data presented in Figures 7 and 8 (see Table S1), 4 independent samples were prepared for each time point. Boxes A–C shown on the 0 hour gel indicate regions of the gel that are shown in enlarged form in Fig. S5.

Sumoylation is a dynamic process, and the sumoylation status of individual proteins is in a constant state of flux depending on the needs of the cell. Our previous study demonstrated changes in expression of the sumoylation components during keratinocyte differentiation, a requirement for active sumoylation in order for differentiation to proceed normally, and changes in the pattern of sumoylated targets that presumably reflected the increased expression of SUMO2/3 as differentiation proceeded [13]. To expand on this previous study we wanted to evaluate dynamic changes in the overall SUMOeome throughout the time course of keratinocyte differentiation. To accomplish this goal we induced SNAP-SUMO3 expression, labeled the SNAP moiety in vivo, and then extracted total protein under highly denaturing conditions to prevent desumoylation of SNAP-SUMO3 modified substrates (Fig. 6A). Samples extracted at 24 hr intervals post-induction of differentiation were concentrated and analyzed directly by 2D gel electrophoresis to evaluate the sumoylation state of proteins over the course of keratinocyte differentiation. A minimum of 4 gels from independent sample preparations were analyzed for each time point, and a representative set of gels (0 to 144 hrs) are shown in Fig. 6B. The SNAP labeling allows direct detection of SNAP-SUMO3 conjugates, and each spot present in these gels is a sumoylated protein. Visual inspection of the gel sets showed excellent uniformity of the spot patterns among the gels for each time point, and consistent landmark patterns between gels for different time points (the overall reduction in signal in the 48 hr sample likely reflects poor in vivo labeling of this one sample as the total protein load was identical for all gels in this series). Boxes A–C in Fig. 6B highlight representative spots showing changes in sumoylation level that are further discussed below. In addition, the fluorescent spot pattern was skewed towards the acidic and higher molecular weight range of the gels compared to the pattern of silver stained total protein, consistent with the charge and mass addition from conjugated SNAP-SUMO (Table 1 and see [18]).

**Table 1:** Relative protein distribution on 2D gels.

Sample	% of Total Spots	
	MW>100 KDa	pI<6.5
Silver Stain (Total Protein)	30%	65%
Fluorescence (Sumoylated Proteins)	67%	75%

**Figure 7:**
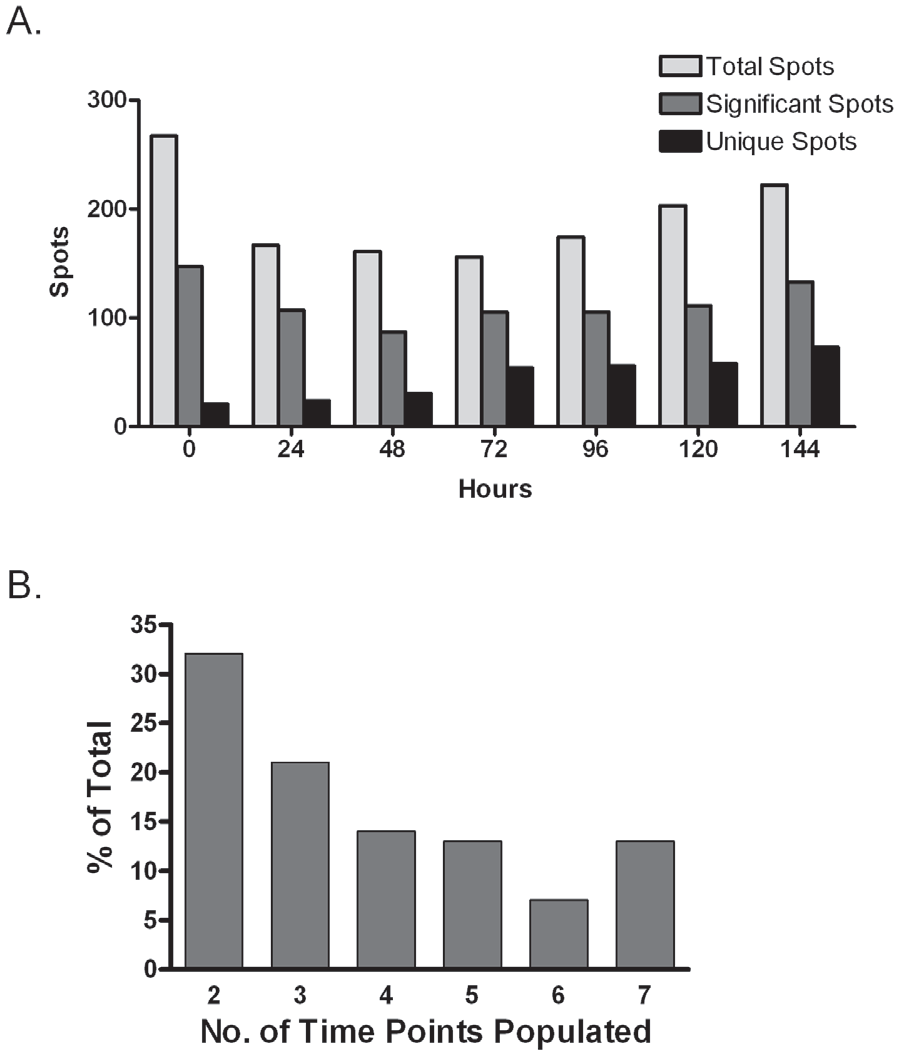
Overall characterization of spot statistics obtained from the 2D analysis. (**A**) The bar graph depicts the number of total detectable spots, statistically significant spots, and unique spots for each time point analyzed. (**B**) A bar graph showing the percentage of spots that were present in from 2–7 time points. Spot data used to derive the graphs in (**A**) and (**B**) are presented in Table S1.

**Figure 8:**
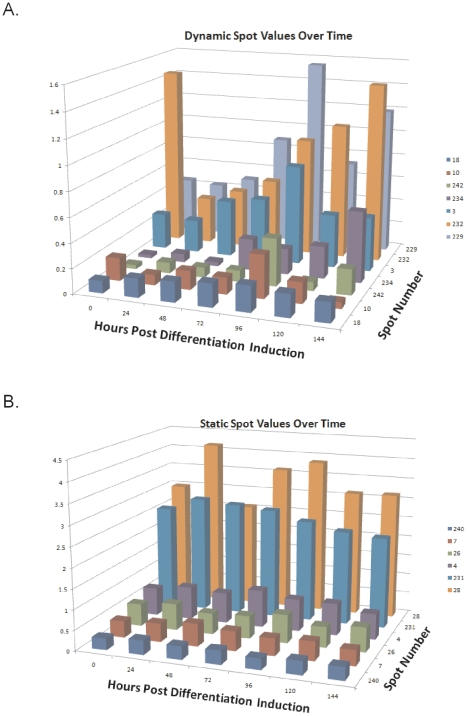
Spot values over the course of differentiation for representative spots. (**A**) A set of bar graphs depicting individual proteins that showed statistically significant changes in spot value over the 0–144 hour period. (**B**) A set of bar graphs depicting representative spots that showed no statistically significant differences during differentiation. Data for both (**A**) and (**B**) are derived from Table S1.

To ascertain the statistical significance of any change in spot values the Melanie 7.0 software package (GeneBio) was used to perform ANOVA and any spots with a p value≤0.05 were considered significant; total spot results are presented in Table S1. Even though spot detection levels were set to a high stringency threshold, the total number of matched spots detected for each set of gels ranged from 156 to 267 (Fig. 7A), indicating a highly complex SUMOeome exists in these cells. The total spot count was greatest in the uninduced (time 0) sample then decreased by 37% over the next 24 hrs as differentiation initiated and major changes in cellular morphology were occurring (see Fig. 4C). Spot number remained relatively constant for the next 48 hrs and then slowly increased after 72 hrs when major changes in differentiation marker expression were manifesting (see Fig. 4A). For each time point, at least 50% of the total spots exhibited statistically significant changes in value over the 6 day period of differentiation. Like the total spot count, the statistically significant spots showed a similar pattern of initial decrease at the onset of differentiation then gradual increase as differentiation proceeded (Fig. 7A). Interestingly, at each time point there was a substantial number of unique spots, and the temporal pattern of the unique spots distribution differed from that of the overall spots. The least number of unique spots was found at time 0, and there was a steady increase in the number of unique spots over the course of differentiation with the largest number found at 144 hours post differentiation. This trend of an increasing number of unique spots over time suggests a changing array of sumoylation targets that parallels the differentiation process and may reflect the biochemical differences between basal and differentiated cells. In addition to the unique spots there were also 126 spots that showed significant changes in value over time but were not unique to one time point; these spots appeared within 2–7 of the time points and the relative distribution of these spot is shown in Fig. 7B. Many spots that appeared only in a limited number of time points did not necessarily do so sequentially (see Table S1), and the basis for this discontinuous expression pattern is unknown. Among the significant spots that were detectable in all time points, their spot values showed a wide variety of changes that were not limited to simple increases (Fig. S5, panel C) or decreases (Fig. S5, panel B), but came in an array of patterns (Fig. 8A). Some spots started off with high values in basal cells that dropped off after differentiation was initiated and then rebounded at a later time point (Fig. 8A, spots 33 and 231). Other spots were low in basal cells and then peaked at an intermediate time before dwindling in late differentiation (Fig. 8A, spots 3 and 228; Fig. S5 panel A). Some spots exhibited relatively small shifts in overall values, but were still considered statistically significant after ANOVA (Fig. 8A, spot 18). In contrast to the spots showing statistically significant changes in value, many spots exhibited consistent values that did not show differentiation-dependent changes (Fig. 8B). The presence of spots with constant values alongside spots that had statistically significant variation in value along with complex expression patterns precludes these spot changes are due to some consistent artifact in the system. Instead, we conclude that the sumoylation of proteins with SUMO3 is a highly dynamic process during keratinocyte differentiation, with many individual target proteins showing discrete changes in their sumoylation status as differentiation progresses. The cadre of proteins showing significant changes in their sumoylation status likely includes proteins that are critical for the propagation of the differentiation program and whose functional activity is being regulated by SUMO addition. Subsequent identification of the significant spots should provide new insight into the critical pathways regulated by sumoylation during keratinocyte differentiation.

## Discussion

Our previous study demonstrated a general increase in sumoylation during keratinocyte differentiation that peaked near 96 hrs and then returned to near basal levels [13]. SUMO1 levels were relatively constant during this period, and the increase in sumoylation appeared largely due to SUMO2/3; as SUMO 2 and 3 are highly homologous we chose SUMO3 for our subsequent studies. To extend our previous observations we wanted to follow a large population of SUMO3 substrates throughout differentiation to examine changes in the sumoylation level of individual proteins. Substrates showing quantitatively significant changes in sumoylation status would typically be ones whose activity was being regulated, and such proteins most likely would be functionally important for progression of the differentiation program. To catalog this group of critical proteins we needed a method to follow a large cohort of individual sumoylated substrates over the multi-day differentiation process, and to that end we developed a novel application of the SNAP-Tag technology coupled with 2D gel analysis. The SNAP-tag is derived from the DNA repair enzyme O^6^-alkylguanine-DNA-alkytransferase and uses derivatives of O^6^-benzylguanine as its fluorescent substrates; these substrates come in cell permeable forms that permit labeling of proteins within the cell. We created a stable HaCaT keratinocyte line containing a single-copy, integrated, tetracycline-inducible SNAP-SUMO3 fusion construct. When induced for expression, the SNAP-SUMO3 protein was effectively conjugated to host proteins and the SNAP-SUMO-substrates were efficiently labeled in vivo with the fluorophore. Since the fluorophore was covalently bound to the SNAP-tag and retained fluorescence under extremely denaturing conditions, cell could be lysed under conditions that inactivated the cellular desumoylating proteases (SENPs) and prevented desumoylation of the modified substrates. The resulting extracts were separated by 2D gel electrophoresis, and the fluorescent SUMO substrates were directly visualized as individual spots that could be accurately quantitated. Other advantages of this approach include: 1) simplicity of sample preparation which reduces biases due to sample loss during purification and allows for feasible analysis of multiple time points and 2) greatly enhanced sensitivity compared to silver staining or immunodetection.

An important concern with this approach was that the HaCaT SNAP-SUMO3 line needed to retain growth and differentiation phenotypes identical to the parental HaCaT line. Examination of growth rate, cell cycle distribution, morphology, and differentiation markers indicated that neither the fusion gene integration nor the expression of the SNAP-SUMO3 protein had any deleterious effect on the biology of this line as long as the SNAP-SUMO induction was for 48 hours or less. Longer induction appeared to lead to senescence, but this phenomenon has not been examined in detail yet. Additionally, fluorescent microscopy of these induced cells showed that SNAP-SUMO3 localized mainly in the nucleus with concentrated labeling at nuclear bodies and with some diffuse labeling in the cytoplasm; which is consistent with published reports regarding SUMO3 localization [7], [22]. We concluded from these result that the HaCaT SNAP-SUMO3 line was equivalent to the parental HaCaT line in terms of growth and differentiation properties and was suitable for detailed examination of sumoylation patterns during differentiation. An additional concern that is more difficult to address is the possibility of artifactual sumoylation of some substrates due to SNAP-SUMO3 levels higher than the physiologic level of endogenous SUMO3. Over expression of sumoylation is a fairly standard approach in sumoylation studies because low endogenous sumoylation levels that often preclude detection of sumoylated species. While we are not aware of any reports that over expression causes widespread artifactual sumoylation, it remains a concern for all studies involving over expression, and our global sumoylation results on the 2D gels will need to be confirmed on an individual substrate basis.

To evaluate the dynamics of sumoylation during differentiation, cultures were induced with calcium and samples were collected at 24 hr intervals for 6 days. The sumoylated proteins in each sample were visualized on 2D gels and quantitated by SNAP fluorescence. ANOVA analysis in triplicate independent experiments identified spots that showed statistically significant changes in sumoylation level. The general trend was that the number of SUMO3 sumoylated proteins decreased rapidly following induction of differentiation followed by a gradual increase over time from 24–144 hrs as the overall number of sumoylated proteins returned to levels similar to those basal cells (time 0). Interestingly, for SUMO3 the general trend in number of spots seemed to follow a somewhat different pattern than observed in our previous report [13] in that we did not previously observe an initial decrease at the start of differentiation. However, our previous study did not distinguish between SUMOs 1, 2, and 3, so the initial decline in SUMO3 modified targets observed here could have been obscured in the previous study by increases in the other SUMOs, particularly SUMO2 as its expression level was the most dramatically increased [13]. It will be important in future studies to evaluate changes in SUMO1 and SUMO2 to determine how those isoforms are each utilized during differentiation.

While overall sumoylation levels followed the general trend of initial decrease followed by a steady increase as differentiation progressed, individual proteins displayed a surprising variety of patterns. At each time point there were a numbers of spots that were uniquely expressed at that time and which were not present in other time points, suggesting highly specific temporal expression or regulation of some substrates. In contrast, other spots persisted throughout differentiation but exhibited diverse patterns of statistically significant increase and/or decrease in sumoylation during the course of differentiation. It is important to note that there were numerous spots present that did not exhibit statistically significant changes and remained constantly expressed from 0–144 hours. These constant spots serve as further proof that the quantitative differences observed in the other spots reflect a genuine difference in biology and not some across the board artifactual change in spot levels at different time points. We conclude that SUMO3 modification is quite complex during keratinocyte differentiation consistent with an important regulatory role in mediating the differentiation program. Note, however, that we cannot attribute all the sumoylation changes to differentiation alone as some may reflect calcium induced effects unrelated to differentiation per se. Clearly identification of specific SUMO substrates and characterization of their biological functions will be necessary to address this question. Finally, we cannot determine from our current study whether the many changes in sumoylated spot intensity are due to changes in abundance of proteins during differentiation or to changes in the sumoylation state for proteins whose quantity remains relatively constant. Both categories would appear biologically important and will be explored in future studies.

In conclusion, we present a new method to observe sumoylation changes using a 2D gel proteomics approach. This study represents the first time an analysis of overall sumoylation has been performed over multiple time point during a differentiation process, and the first time SNAP-tag technology has been used for in gel detection of sumoylated proteins. This method can be applied to other ubiquitin like modifiers and is an excellent means of comparing conditional and dynamic sumoylation between samples over extended periods of time as it allows direct visualization of sumoylated proteins with minimal sample processing. This powerful technology has revealed that the sumoylation of proteins during keratinocyte differentiation is a highly dynamic and diverse process with numerous proteins exhibiting extensive changes in their sumoylation state. The relevant proteins showing significant changes in sumoylation state have been cataloged and future goals include identifying these proteins so that role of sumoylation in their individual contribution to keratinocyte differentiation can be assessed.

## Materials and Methods

### Cell culture conditions

The HaCaT parental cell line [13] and the HaCaT FRT/TR#8 derivative were maintained in Dulbecco's Minimum Eagle Medium supplemented with 10% Benchmark FBS (Gemini Bio), 4 mM glutamine, and either 0.03 mM calcium (low) or 2.38 mM calcium (high) dependent on induction conditions. The HaCaT SNAP-SUMO3 cells were maintained in similar conditions with the exception of substituting 10% tetracycline screened FBS (HyClone) in place of Benchmark FBS. Media for HaCaT FRT/TR#8 cultures were supplemented with 100 µg/ml zeocin and 10 µg/ml blasticidin, while media for HaCaT SNAP-SUMO3 cells were supplemented with 100 µg/ml hygromycin and 10 µg/ml blasticidin. To induce the expression of SNAP-SUMO3 cells tetracycline was added to media at a concentration of 1 µg/ml.

### Creation of a stable SNAP-SUMO3 cell line

Generation of HaCaTs expressing the SNAP-tagged SUMO was done using the Invitrogen Flp-In T-REx system according to the manufacturer's directions. Briefly, HaCaTs were transfected with the pFRT/lacZeo plasmid and cells containing the integrated plasmid were selected using 100 µg/ml of zeocin. Resistant colonies were screened for the ability to express high levels of β-galactosidase) and for the presence of a single copy of the integrated pFRT/lacZeo plasmid (using Southern Blot analysis; data not shown). Several clonal lines were subsequently transfected with a plasmid encoding the Tet repressor, pcDNA6/TR, and selected with 100 µg/ml of zeocin and 10 µg/ml of blasticidin. Those clones that expressed the most Tet repressor, as determined by inhibition of β-galactosidase expression, were expanded and one clone was chosen as the parental cell line designated HaCaT FRT/TR#8.

The SNAP tag sequence was PCR amplified from the pSS26m mammalian source plasmid (Covalys) using forward (5′-ATCGATAAGCTTGATATCACCATGGACAAAGACTGC -3′; Tm 62.2°C) and reverse primers (5′- TATAAGCTTGCCCAGCCCAGGCTTGCCCAGTC-3′; Tm 69.9°C). The resulting PCR product was gel purified (Qiagen), cloned into the pGem T Easy vector (Promega) using HindIII sites introduced by the primers, and transformed into chemically competent DH5α cells. The resulting pGemT-SNAP plasmid was cloned into the SUMO3 expression vector, pcDNA5FRT/TO/His-S-SUMO3, via the HindIII sites. Plasmid DNAs from the resulting colonies were screened for the insert by digestion with HindIII (NEB). Colonies that were positive for the insert were then sequenced with forward (5′-GAAAACCGCCCTGAGCGGAAATCC-3′; Tm 62.6°C) and reverse primers (5′-TCGCACCCAGACAGTTCCAGCTT-3′; Tm 62.9°C) to ensure proper orientation of the SNAP-tag. A plasmid exhibiting proper sequence and orientation was designated pcDNA5FRT/TO/SNAP-His-S-SUMO3 and was used to transform DH5α. Final plasmid DNA was prepared by a maxiprep and was further purified via a cesium chloride gradient.

The parental cell line, HaCaT FRT/TR #8, was plated at 30% confluency on 10 cm tissue culture plates and allowed to adhere overnight at 37°C and 5% C0_2_. The following day 2.6 µg of pcDNA5FRT/TO/SNAP-His-S-SUMO3 was transfected with 21.4 µg of the Flp recombinase plasmid pOG44 via Lipofectamine 2000 (Invitrogen). The cells containing the inserted cassette were selected with hygromycin (100 µg/ml) and blasticidin (10 µg/ml) added three days after transfection to allow expression of the antibiotic resistance genes. After two weeks of selection cells were pooled and stocks were frozen in liquid nitrogen or used for further experiments.

### Growth curve and cell cycle distribution

HaCaT FRT/TR and HaCaT SNAP-SUMO3 cells were each plated to give 1.0×10^5^ cells per 60 mm dish. At 24 hours after plating (time 0), half the dishes in each set were treated with tetracycline at 1 ug/ml to induce SNAP-SUMO3 and the other half were left as uninduced. Starting at time 0 and then at 24 hr. intervals, 3 dishes from both the induced and uninduced sets were trypsinized and counted to determine the growth rate of the cells. Medium and tetracycline was changed daily for the remaining plates to ensure optimal induction of SNAP-SUMO3. To determine the effect of SNAP-tagged SUMO3 expression on cell cycle distribution, 4.5×10^5^ parental and SNAP-SUMO3 expressing HaCaTs were plated into induced and uninduced groups and allowed to adhere overnight. The following day SNAP-SUMO3 expression was induced with 1.0 µg/ml tetracycline; after 24 hours fresh medium and tetracycline were changed to ensure maximum SUMO3 expression. Following 48 hr. of induction, the cells were trypsinized, washed, fixed with 60% ethanol overnight at 4°C, and then stained with PI staining solution containing 50 µg/ml propidium iodide, 4 mM sodium citrate, 5 mM EDTA, 0.1% Triton X-100, and 30 U/ml of RNAse I. Cells were then subjected to flow cytometry analysis using a FACSCalibur (Becton Dickinson), and the results were analyzed with ModFit LT (Verity Software House), and graphed using GraphPad Prism 4.0.

### Microscopy and imaging

For phase and fluorescent images the HaCaT and HaCaT SNAP-SUMO3 cells were plated at 1.5×10^5^ cells per 60 mm tissue culture dish and SNAP-SUMO3 was induced for 48 hrs with 1 µg/ml tetracycline. For phase microscopy the cells were imaged using a DP71 camera with an Olympus IX81 microscope using the DP71 controller software; images of the cells were captured after 8 ms exposure using 200× magnification. Fluorescent images were captured after cells were labeled with SNAP-Cell Fluorescein (NEB) per the manufacturer's instructions. The cells were exposed for 20 ms and images were captured using the Olympus IX81 microscope with the DP71 camera and FITC filter set at 20× magnification. All images were exported to GraphPad Prism.

### Western blot procedure

Western Blots were done to assess the expression of K1, involucrin and loricrin as well as the expression of SNAP-tagged SUMO. In all cases, tubulin was used as a loading control. For evaluation of the differentiation markers (K1, involucrin and loricrin), cells were plated on 60 mm plates so that at each time point the cells would be 80–85% confluent. To optimize the concentration of tetracycline needed for induction of SNAP-tagged SUMO, 4.0×10^5^ cells were plated and allowed to adhere overnight. The next day tetracycline was added to the cells at 0, 0.5, 1.0, 2.0, and 4.0 µg/ml and induction was carried out for 24 or 48 hrs with the media and tetracycline being changed every 24 hrs for maximum induction. Cells were washed twice in 1× PBS, lysed in boiling 4× sample buffer (100 mM Tris pH 6.8, 20% glycerol, 8% SDS, 0.02% bromophenol blue, and 4% β-mercaptoethanol), and then passed through a 27 gauge needle ten times. Samples were subsequently loaded onto either 8% (K1, involucrin, loricrin, and tubulin) or 6% (S tag) polyacrylamide gels and run at 90 V until exiting the stacking gel and then 110 V for the resolving gel. Following electrophoresis, protein was transferred to 0.2 µm PVDF membranes at 1.2 Amps, constant amperage. Membranes were blocked with 5% nonfat dried milk (NFD) for thirty minutes followed by probing with primary antibody. The following antibody dilutions were used: 1∶1,000 for K1, 1∶500 for Loricrin, 1∶1,000 for Involucrin, and 1∶1,000 for tubulin. Secondary antibodies were used at a concentration of 1∶5,000. SNAP-SUMO3 was detected using S protein conjugated to HRP at a 1∶500 dilution or using anti-SUMO2/3 (Santa Cruz) at a 1∶250 dilution. Detection of the S protein conjugate was done with the Millipore Immobilon Chemiluminescent HRP Detection kit, and the detection for the anti-SUMO3 blots was done with the Super Signal West Pico kit (Pierce). Images for both types of blots were captured using the Alpha Innotech imager.

### 2D gel electrophoresis of proteins

Cells were plated so that at each time point the cells would be about 80% confluent. Cells were maintained in DMEM with 0.03 mM calcium supplemented with 10% tetracycline screened FBS and 100 µg/ml hygromycin and 10 µg/ml blasticidin. To induce differentiation the calcium concentration was raised to 2.3 mM. For each time point, tetracycline was added 48 hours prior to cell harvest at a final concentration of 1.0 µg/ml to induce SNAP-SUMO3 expression. Fresh medium and tetracycline were added at 24 hours prior to harvest to ensure continued expression of the SNAP-SUMO3 during this pre-collection phase. Cells were harvested by washing twice with cold 1× PBS and lysed in 2D sample prep buffer containing 8M urea, 4% CHAPS, 0.5% Pharmalytes pH 3–10, and 40 mM DTT. Samples were spun at 13,000 RPM for two minutes to pellet cellular membranes and debris. Protein concentrations were calculated using the 2-D Quant Kit (GE Life Sciences) per the manufacturer's directions. To concentrate and clean the samples, aliquots containing 200 µg of protein were methanol-chloroform precipitated, and the pellet was resuspended in 150 µl of 2D sample prep solution. The samples were then cup loaded onto pH 3–11 NL strips that were rehydrated overnight in rehydration buffer containing 8M urea, 2% CHAPS, 0.5% Pharmalytes, and 0.002% bromophenol blue. The first dimension was run on an Ettan IPGphor3 as follows: STP 500 V for 3 hrs, GRD 1000 V for 1 hr, GRD 8000 V for 2.5 hr, and STP 8000 V for 0.5 hr for a total of 13,000 Vhrs on average. After isoelectric focusing the strips were equilibrated in SDS equilibration buffer containing 6 M urea, 75 mM Tris-HCl pH 8.8, 29.3% glycerol, 2% SDS, 0.002% bromophenol blue and 10 mg/ml DTT, followed by equilibration in SDS equilibration buffer without DTT and supplemented instead with 25 mg/ml iodoacetamide. For the second dimension, the strips were placed on top of a 6% polyacrylamide gel, sealed with an agarose solution containing SDS running buffer (25 mM Tris base, 192 mM Glycine, 0.1% SDS) 0.5% agarose, and 0.002% bromophenol blue, and resolved overnight at 15 Amps.

### Imaging and analysis of gels

2D gels were imaged with the Typhoon Trio variable mode imager using the 532 nm laser and the 580 nm bp filter at 800 PMT and high sensitivity setting. 2D gels of SNAP-labeled samples were analyzed using the Melanie 7 software (GeneBio). Gels were separated into classes based on the time the samples were taken post calcium induced differentiation. Spots were detected with the following settings: Smooth: 2, Saliency: 50, and Min Area: 100. Gels were visually scrutinized to ensure the accuracy of spot detection with spots added or deleted as needed. Spots were matched within each match set and then between the classes. Matched gels were then visually scrutinized to ensure the accuracy of the matches. Inaccurate matches were corrected by breaking and remaking matches. To determine differences in protein levels between the time points ANOVA analysis was performed between the classes. Any spot with a p value≤0.05 was considered significant.

## Supporting Information

Figure S1 **Cell cycle histograms for HaCaT and HaCaT SNAP-SUMO3 cells.** Cells were prepared and analyzed for DNA content as described in Materials and Methods. Shown are the histograms for one of the sample sets used to derive the data in Figure 3A. Cultures designated as induced in the figure were treated with tetracycline.
(TIF)

Figure S2 **SNAP-SUMO3 induces senescence in HaCaT cells.** SNAP-SUMO3 HaCaT cells were plated and left uninduced (A) or SNAP-SUMO3 production was induced with tetracycline and cells were harvested at 24 hrs (not shown), 48 hrs (B), or 72 hours (C). Fibroblasts were plated and mock infected (D; Uninfected) or infected with the Towne strain of HCMV at an MOI of 5 (E; CMV-Infected). At the indicate times post induction or infection all the cultures were stained for senescence associated β-galactosidase activity.
(TIF)

Figure S3 **Keratin 1 (K1) induction kinetics in HaCaT and HaCaT SNAP-SUMO3 cells.** Parallel cultures of HaCaT and HaCaT SNAP-SUMO3 cells were placed into high calcium medium at time 0 and cultured for 6 days. At 24 hr intervals cells were harvested and extracts were immunoblotted with anti-keratin 1 and anti-tubulin.
(TIF)

Figure S4 **HaCaT SNAP-SUMO3 cells display normal localization of SUMO3.** (A–C) SNAP-SUMO3 HaCaT cells were induced with tetracycline for SNAP-SUMO3 production for 48 hours. At 48 hours post induction the SNAP-SUMO3 nuclei were detected with Hoechst stain (A) and SNAP-SUMO3 was labeled with SNAP-Cell 505 and visualized by fluorescent microscopy (B). (C) Overlay of the images shows primarily nuclear localization with some cytoplasmic staining. (D–F) Uninduced SNAP-SUMO3 HaCaT cells were stained with DAPI (D) or endogenous SUMO2/3 was visualized with anti-SUMO2 (E), and the overlay is shown in (F). (G–I) Parental HaCaT cells treated as in D-F, respectively.
(TIF)

Figure S5 **Time course of changes in representative individual 2D spots.** Regions A, B, and C indicated on the zero hour 2D gel in Fig. 6B were captured and enlarged for 5 time points (0, 24, 72, 96, and 144 hrs). The red and blue boxes within panel A show 2 spot sets that appear as differentiation initiates and then diminish as the fully differentiated state is reached by 144 hrs. Panels B and C show spots that decrease or increase, respectively, as differentiation proceeds.
(TIF)

Materials and Methods S1 (DOCX)

Table S1 (XLS)
