# Supplementary figures and images for: Analysis of Global Sumoylation Changes Occurring during Keratinocyte Differentiation
Source: PLoS One. 2012 Jan 23;7(1):e30165. doi: 10.1371/journal.pone.0030165 (PMC3264615; doi:10.1371/journal.pone.0030165)

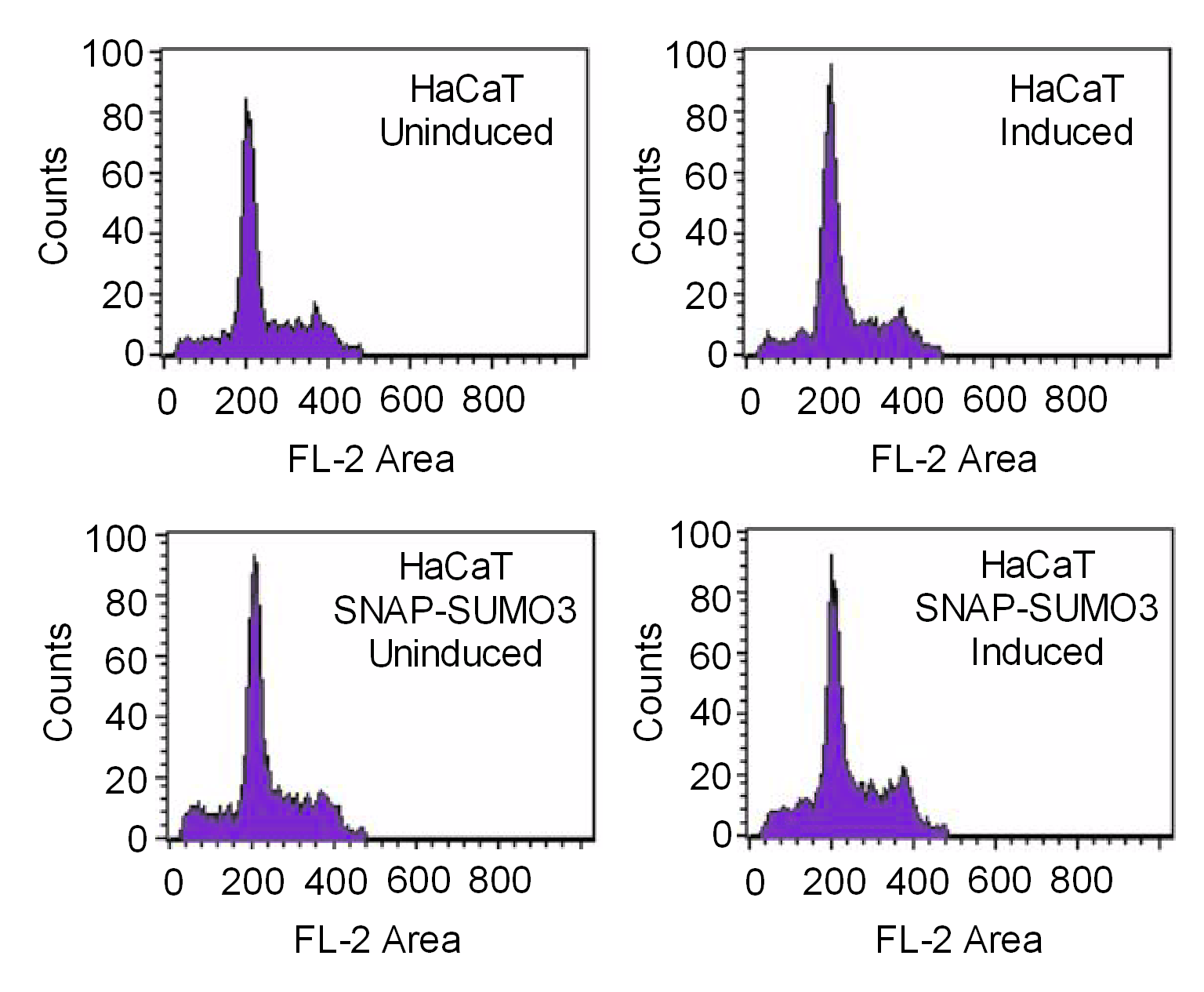

Supplement: Figure S1 — Cell cycle histograms for HaCaT and HaCaT SNAP-SUMO3 cells. Cells were prepared and analyzed for DNA content as described in Materials and Methods. Shown are the histograms for one of the sample sets used to derive the data in Figure 3A. Cultures designated as induced in the figure were treated with tetracycline. (TIF) [file pone.0030165.s001.tif]

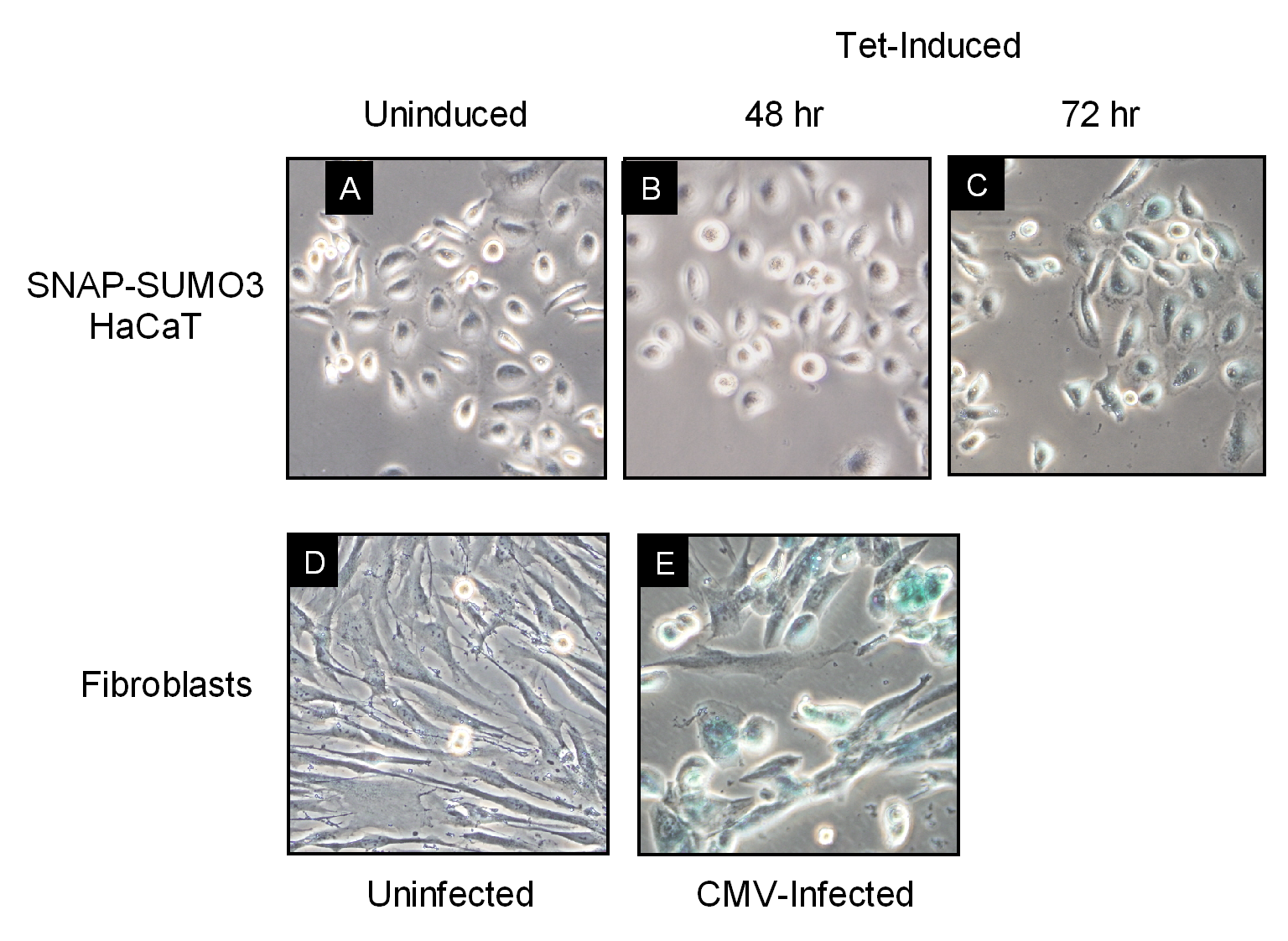

Supplement: Figure S2 — SNAP-SUMO3 induces senescence in HaCaT cells. SNAP-SUMO3 HaCaT cells were plated and left uninduced (A) or SNAP-SUMO3 production was induced with tetracycline and cells were harvested at 24 hrs (not shown), 48 hrs (B), or 72 hours (C). Fibroblasts were plated and mock infected (D; Uninfected) or infected with the Towne strain of HCMV at an MOI of 5 (E; CMV-Infected). At the indicate times post induction or infection all the cultures were stained for senescence associated β-galactosidase activity. (TIF) [file pone.0030165.s002.tif]

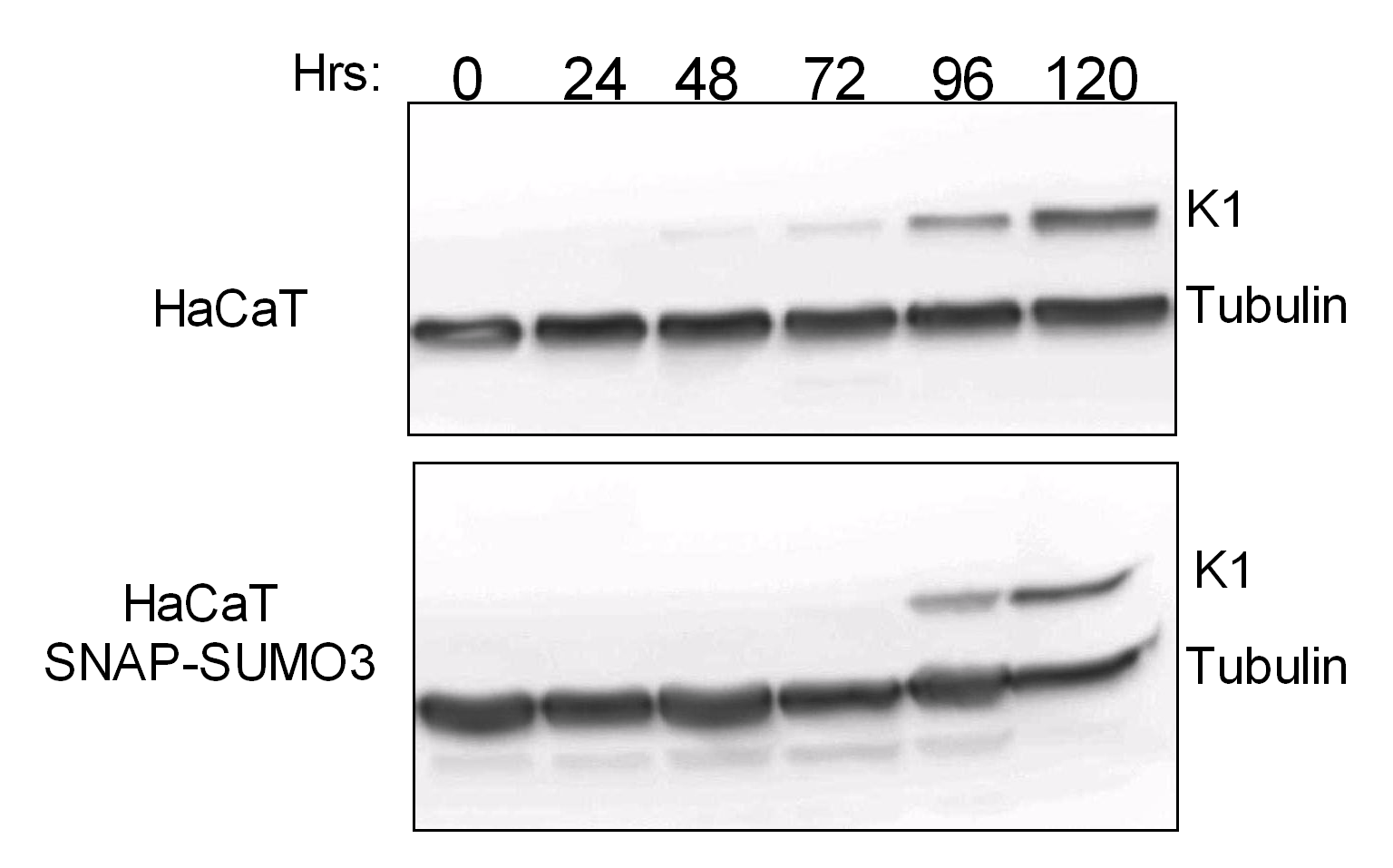

Supplement: Figure S3 — Keratin 1 (K1) induction kinetics in HaCaT and HaCaT SNAP-SUMO3 cells. Parallel cultures of HaCaT and HaCaT SNAP-SUMO3 cells were placed into high calcium medium at time 0 and cultured for 6 days. At 24 hr intervals cells were harvested and extracts were immunoblotted with anti-keratin 1 and anti-tubulin. (TIF) [file pone.0030165.s003.tif]

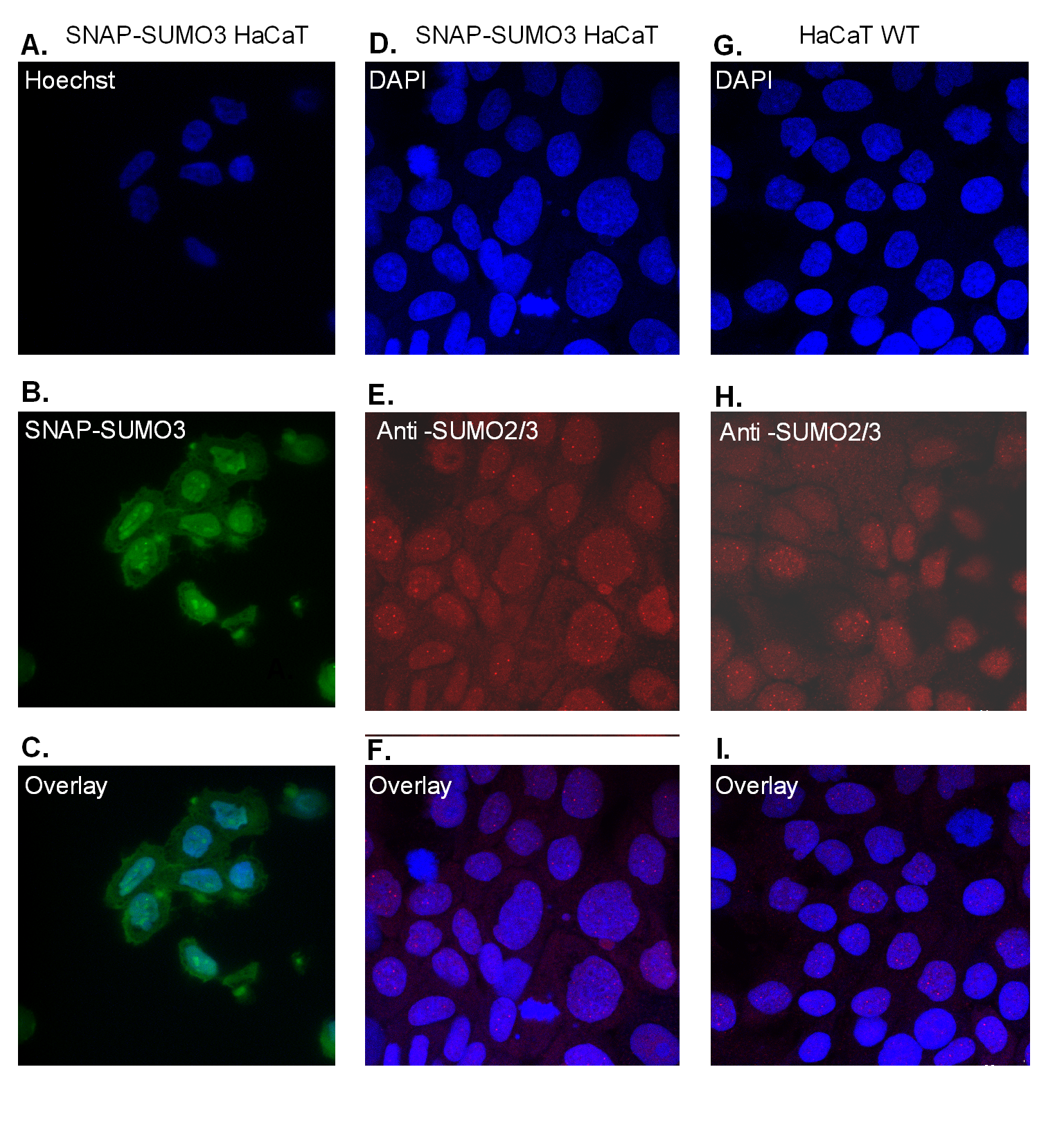

Supplement: Figure S4 — HaCaT SNAP-SUMO3 cells display normal localization of SUMO3. (A–C) SNAP-SUMO3 HaCaT cells were induced with tetracycline for SNAP-SUMO3 production for 48 hours. At 48 hours post induction the SNAP-SUMO3 nuclei were detected with Hoechst stain (A) and SNAP-SUMO3 was labeled with SNAP-Cell 505 and visualized by fluorescent microscopy (B). (C) Overlay of the images shows primarily nuclear localization with some cytoplasmic staining. (D–F) Uninduced SNAP-SUMO3 HaCaT cells were stained with DAPI (D) or endogenous SUMO2/3 was visualized with anti-SUMO2 (E), and the overlay is shown in (F). (G–I) Parental HaCaT cells treated as in D-F, respectively. (TIF) [file pone.0030165.s004.tif]

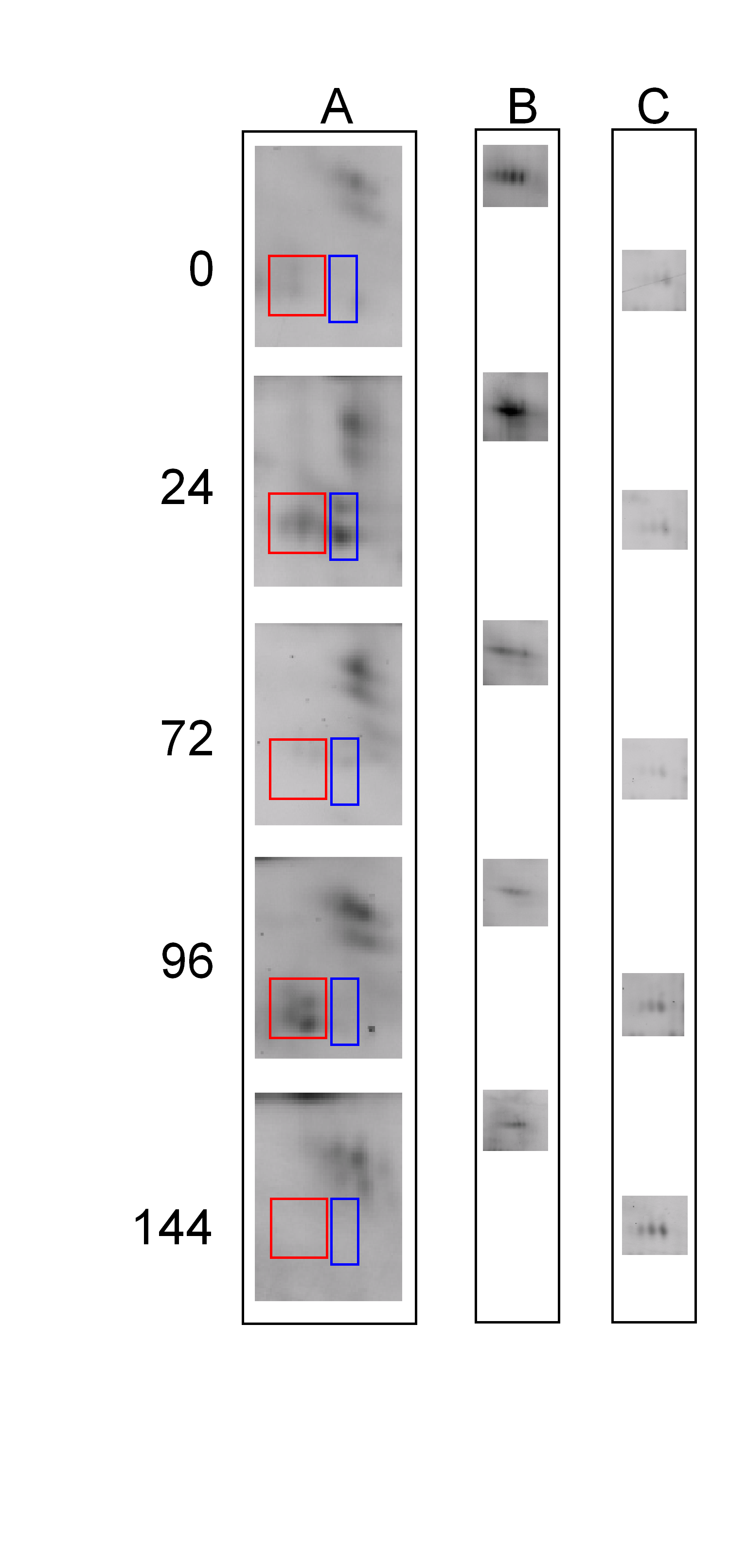

Supplement: Figure S5 — Time course of changes in representative individual 2D spots. Regions A, B, and C indicated on the zero hour 2D gel in Fig. 6B were captured and enlarged for 5 time points (0, 24, 72, 96, and 144 hrs). The red and blue boxes within panel A show 2 spot sets that appear as differentiation initiates and then diminish as the fully differentiated state is reached by 144 hrs. Panels B and C show spots that decrease or increase, respectively, as differentiation proceeds. (TIF) [file pone.0030165.s005.tif]
