# Supplementary material for: Analysis of Global Sumoylation Changes Occurring during Keratinocyte Differentiation
Source: PLoS One. 2012 Jan 23;7(1):e30165. doi: 10.1371/journal.pone.0030165 (PMC3264615; doi:10.1371/journal.pone.0030165)
Supplement: Materials and Methods S1 — (DOCX) [file pone.0030165.s006.docx]

SUPPLEMENTAL METHODS

Senescence Associated β-gal Assay

SNAP-SUMO3 HaCaTs were plated at 2.0x10^5^ cells per 60 mm dish and human fibroblasts were plated at 3.0x10^5^ (mock infected) and 1.0x10^6^ (HCMV infected) cells per 60 mm tissue culture dish. Fibroblasts were either mock infected or infected at an MOI of 5 with the Towne strain of and grown for 72 hours. SNAP-SUMO3 was induced for 72 hours with the media and tetracycline being changed every 24 hours. At 24 hr intervals cells were washed three times with PBS and fixed with 2% formaldehyde and 0.2% glutaraldehyde for 5 minutes. Cells were then washed with PBS and stained for 12 hours with a staining solution containing 5 mM K_3_FECN_6_, 2 mM MgCl_2_, 150 mM NaCl, 30 mM citric acid/phosphate buffer, 5 mM K_4_FECN_6_, and 1 mg/ml X-Gal. Cells were visualized using an Olympus IX81 microscope with a DP71 camera.

Confocal Microscopy

For visualization of SNAP-SUMO3, 1.5 x 10^5^ HaCaT SNAP-SUMO3 cells were plated and allowed to adhere overnight. The following day SNAP-SUMO3 production was induced with tetracycline and was continued for 48 hours with fresh media and tetracycline added at 24 hours. After 48 hours of induction the cells were labeled for 1 hour with SNAP-Cell 505. Cells were then fixed in 2% formaldehyde for 20 minutes and stained with Hoechst stain for twenty minutes. Cells were visualized with an Olympus IX81 microscope equipped with a DSU spinning disk confocal unit with a Retiga-SRV Fast 1394 monochromatic CCD camera. Images were captured sequentially with an exposure time of 100ms for DAPI and 2000 ms for FITC with a step size of 0.5 micron. For visualization of endogenous SUMO2/3, parental HaCaT or HaCaT SNAP-SUMO3 cells were seeded at a cell density of 1.5X10^4^ in a flat bottom 96 well black polypropylene plate (Whatman Piscataway, NJ). After 48h, the cells were fixed for 30 minutes in 1 x phosphate buffered saline (PBS) supplemented with 4% Paraformaldehyde. Subsequently, the cells were washed twice with 1 x PBS supplemented with 10% goat serum and permeabilized with methanol for 10 minutes. The cells were washed twice with 1 x PBS + 10% goat serum and incubated for 6 hr with anti-SUMO2 rabbit polyclonal antibodies (Invitrogen Corp. Cat#519100) at a 1:100 dilution in the wash buffer. After primary serum incubation the cells were washed three times with 1 x PBS + 10% goat serum and incubated for 2 hr with Alexa Fluor® 594 Goat Anti-Rabbit IgG (H+L) *Highly Cross-Adsorbed (Invitrogen Corp. Cat#A11037) at a 1:500 dilution. Finally, the cells were washed three times with 200 ml of 1 x PBS + 10% goat serum, then ten minutes before analyzing the plate a final concentration of 5 mg/mL of DAPI was added to stain the nucleus. The images were captured by using an LSM 700 confocal microscope equipped with monochromatic camera (Zeiss.  New York, NY), a 63X objective and two lasers (405 nm for DAPI and 555 nm for Alexa), and the ZEN 2009 software (Zeiss, New York, NY) which is designed for acquisition and processing of confocal images. Computerized pseudo color was added to differentiate the various fluorescence channels and achieve the final colored images.

Supplemental Figure Legends

Fig. S1. **Cell cycle histograms for HaCaT and HaCaT SNAP-SUMO3 cells.** Cells were prepared and analyzed for DNA content as described in Materials and Methods. Shown are the histograms for one of the sample sets used to derive the data in Figure 3A. Cultures designated as induced in the figure were treated with tetracycline.

Fig. S2. **SNAP-SUMO3 induces senescence in HaCaT cells**. SNAP-SUMO3 HaCaT cells were plated and left uninduced (A) or SNAP-SUMO3 production was induced with tetracycline and cells were harvested at 24 hrs (not shown), 48 hrs (B), or 72 hours (C). Fibroblasts were plated and mock infected (D; Uninfected) or infected with the Towne strain of HCMV at an MOI of 5 (E; CMV-Infected). At the indicate times post induction or infection all the cultures were stained for senescence associated β-galactosidase activity.

Fig. S3. **Keratin 1 (K1) induction kinetics in HaCaT and HaCaT SNAP-SUMO3 cells.** Parallel cultures of HaCaT and HaCaT SNAP-SUMO3 cells were placed into high calcium medium at time 0 and cultured for 6 days. At 24 hr intervals cells were harvested and extracts were immunoblotted with anti-keratin 1 and anti-tubulin.

Fig. S4. **HaCaT SNAP-SUMO3 cells display normal localization of SUMO3.** (A-C) SNAP-SUMO3 HaCaT cells were induced with tetracycline for SNAP-SUMO3 production for 48 hours. At 48 hours post induction the SNAP-SUMO3 nuclei were detected with Hoechst stain (A) and SNAP-SUMO3 was labeled with SNAP-Cell 505 and visualized by fluorescent microscopy (B). (C) Overlay of the images shows primarily nuclear localization with some cytoplasmic staining. (D-F) Uninduced SNAP-SUMO3 HaCaT cells were stained with DAPI (D) or endogenous SUMO2/3 was visualized with anti-SUMO2 (E), and the overlay is shown in (F). (G-I) Parental HaCaT cells treated as in D-F, respectively.

Fig. S5. **Time course of changes in representative individual 2D spots.** Regions A, B, and C indicated on the zero hour 2D gel in Fig. 6B were captured and enlarged for 5 time points (0, 24, 72, 96, and 144 hrs). The red and blue boxes within panel A show 2 spot sets that appear as differentiation initiates and then diminish as the fully differentiated state is reached by 144 hrs. Panels B and C show spots that decrease or increase, respectively, as differentiation proceeds.
